# Effects of scaling direction on adults’ spatial scaling in different perceptual domains

**DOI:** 10.1038/s41598-023-41533-3

**Published:** 2023-09-06

**Authors:** Magdalena Szubielska, Marta Szewczyk, Paweł Augustynowicz, Wojciech Kędziora, Wenke Möhring

**Affiliations:** 1 Faculty of Social Sciences, Institute of Psychology, The John Paul II Catholic University of Lublin, Lublin, Poland; 2 Unaffiliated, Lublin, Poland; 3 Faculty of Psychology, University of Basel, Basel, Switzerland; 4 Department of Educational and Health Psychology, University of Education Schwäbisch Gmünd, Schwäbisch Gmünd, Germany

**Keywords:** Psychology, Human behaviour

## Abstract

The current study investigated adults’ strategies of spatial scaling from memory in three perceptual conditions (visual, haptic, and visuo-haptic) when scaling up and down. Following previous research, we predicted the usage of mental transformation strategies. In all conditions, participants (*N* = 90, aged 19–28 years) were presented with tactile, colored graphics which allowed to visually and haptically explore spatial information. Participants were first asked to encode a map including a target. Then, they were instructed to place a response object at the same place on an empty, constant-sized referent space. Maps had five different sizes resulting in five scaling factors (3:1, 2:1, 1:1, 1:2, 1:3). This manipulation also allowed assessing potentially symmetric effects of scaling direction on adults’ responses. Response times and absolute errors served as dependent variables. In line with our hypotheses, the changes in these dependent variables were best explained by a quadratic function which suggests the usage of mental transformation strategies for spatial scaling. There were no differences between perceptual conditions concerning the influence of scaling factor on dependent variables. Results revealed symmetric effects of scaling direction on participants’ accuracy whereas there were small differences for response times. Our findings highlight the usage of mental transformation strategies in adults’ spatial scaling, irrespective of perceptual modality and scaling direction.

## Introduction

Spatial scaling is an essential spatial skill that is involved in several daily activities. It is fundamental for various professions and is a crucial prerequisite for several science disciplines^1–4^. Spatial scaling ability involves a comparison of different-sized spaces and an understanding of the spatial relations between them^5^. In a typical spatial scaling task, participants are presented with a simple map including a target and an empty referent space and asked to locate the target in the referent space^3,5–12^. By systematically varying sizes of one space while keeping the other one constant, participants need to scale spatial information from one space to the other. Using this kind of mapping task, it was shown that the ability to scale spatial information seems to emerge early in the life course^6^. Furthermore, scaling seems to develop considerably across childhood^5,7,9,12^, but yet shows variability in adulthood^8,10,11^.

### Spatial scaling strategies

**Figure 1:**
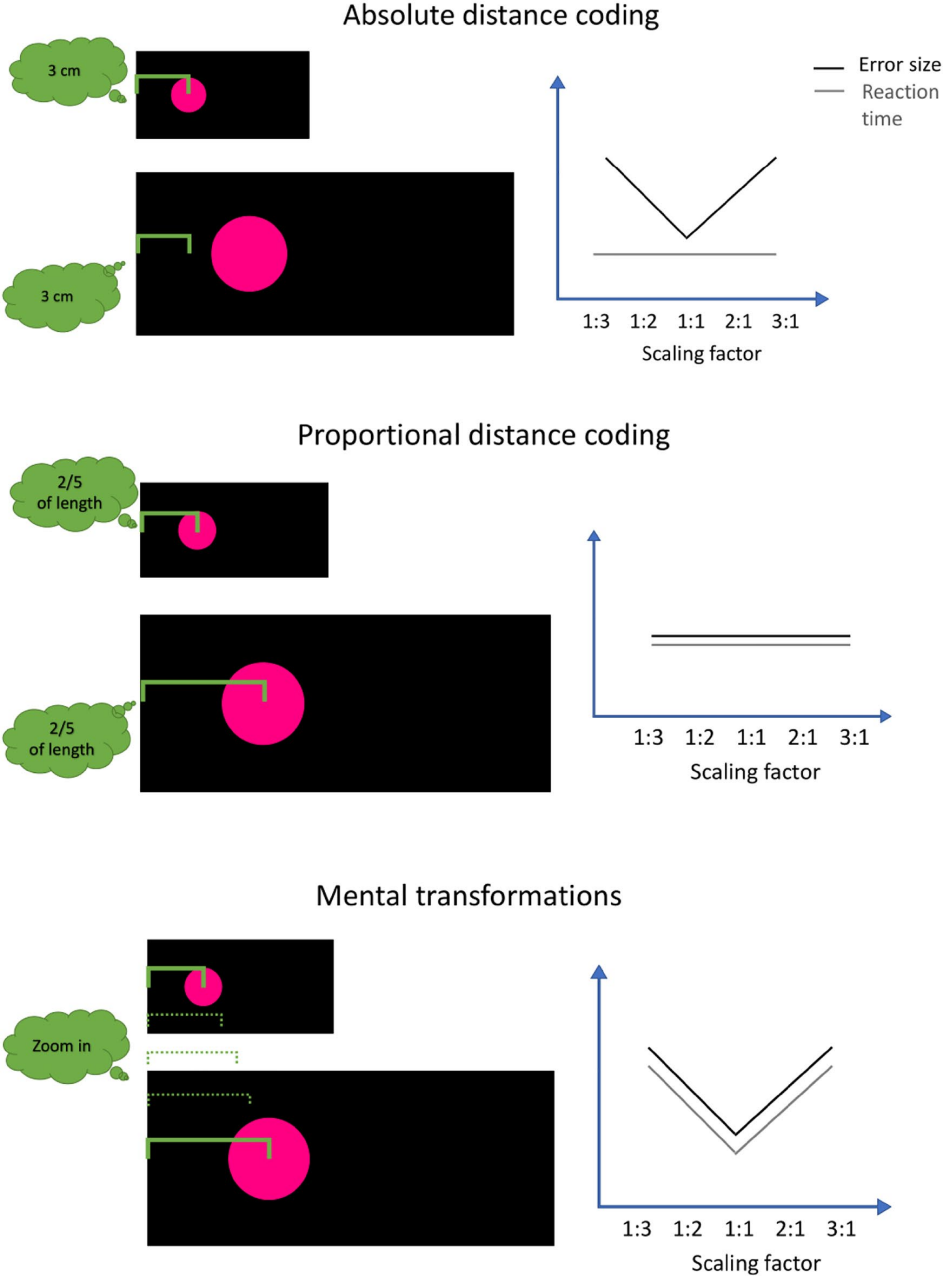
Effects of different spatial scaling strategies on participants’ accuracy and response times.

Three different strategy types have been distinguished in the previous literature in order to solve such a spatial scaling task^13,14^ (see Fig. 1). In the first strategy, participants may encode the spatial information provided in a map and match the identical information onto a given referent space in an absolute way, i.e., ignoring potential differences in the sizes of the spatial layouts. Therefore, with higher scaling factors between the map and the referent space, errors would increase. However, such an absolute strategy would not affect participants’ response times. A second strategy relates to encoding spatial information in a proportional manner. Using this strategy, participants may encode relative distances (i.e., a target being one-fifth from the left landmark) and map this information on a referent space^6,15^. Regardless of whether the map and the referent space differ in size, this strategy would work accurately and quickly. Consequently, participants’ errors and response times may remain constant across different scaling factors. A third strategy relates to employing mental transformation strategies (also known as mental zooming). Evidence for mental transformations has been found in research on mental rotation, scanning, and object matching^16–19^. In particular, participants may encode spatial information in the map and re-size it using mental imagery^20^. By doing so, they may transform this spatial information while taking size differences between the map and the referent space into account. In line with findings from mental imagery research^18,19^, these processes may take longer and be more error-prone with larger transformations. Hence, when using mental zooming, response times and errors may increase in a linear way with higher scaling factors.

A crucial precondition for differentiating spatial scaling strategies is to systematically manipulate the size difference between the map and the referent and, therefore, to use various scaling factors. Furthermore, it is fundamental to measure errors *and* response times considering that strategies are associated with a differential pattern of these two dependent variables^20,21^. Previous results of spatial scaling research that adopted a methodology consistent with the aforementioned preconditions suggested the usage of mental transformation strategies in the visual domain^8,14^ and haptic domain^20,21^.

### Effects of perceptual modality

Spatial information can be perceived across the visual and haptic sense. Perceiving objects by touch provides—similar to the visual domain—information about an object’s linear size, orientation, shape, texture, and spatial relations. By contrast to vision that allows to quickly perceive a relatively large spatial area^22^ and to process a complex image^23^, haptic exploration does not allow to perceive all the objects in a spatial layout at once^24,25^. Instead, the observer has to serially explore the object’s relation to other objects which takes more time and is limited to the observer’s peripersonal space (i.e., the space around the body).

Previous research that compared participants’ recognition of maps or objects in the visual and haptic domain revealed similarities between performances in these modalities^21,26–30^. That is, participants showed comparable response times and errors in both domains^28^ and performance was similarly affected by size changes in each of these modalities^21,26–28^. Moreover, recent studies have found that a phenomenon called visual canonical size^31^ can be observed in drawings made under visual and haptic control^32–34^. This phenomenon goes back to Konkle and Olivia^31^ who assumed that mental object representations comprise size information of real-world objects consistently because humans view objects at various visual angles (more vs. less canonical ones). However, the nature of the phenomenon was recently questioned given that drawings produced in the visual and haptic domain increased in size, when larger real-world objects were depicted^32–34^. Summing up, these findings are in line with the hypothesis of functional equivalence of spatial representations from touch and vision^28,30,35^.

But there are also studies showing that performance differed between the haptic and visual domain. For example, in a recent study^22^, participants were asked to estimate a spatial area that was learned either through the visual or haptic sense. It was found that participants overestimated this spatial area to a larger extent in the visual condition as compared to the haptic perceptual condition (a phenomenon called boundary extension). Therefore, it seems that perceiving spatial information via the haptic sense can—on some occasions—trump the visual sense. On the contrary, another recent study showed that spatial performance might be more accurate for the visual as compared to the haptic perceptual condition. In this respective spatial scaling study, participants were instructed to explore a three-dimensional object by vision or touch and then to imagine it in a given scale and estimate its size^36^. Results indicated that adults performed more accurately in the visual than haptic condition. This higher accuracy in the visual domain is supported by research on cognitive map formation showing that complex city-like maps were more accurately explored through visual than haptic exploration^30^ (a similar effect did not occur in the case of less complex maps). Furthermore, in previous studies on spatial scaling, participants located targets on the referent space less correctly in the haptic than both in the visual condition or the bimodal (visuo-haptic) condition^21,37^.

### Effects of scaling directions: up versus down

There is a lack of research examining the effects of scaling direction, especially in the haptic domain. Children and adults are frequently confronted with both scaling directions in their daily life, such as when scaling up spatial information provided in a map to the real-world location, they are navigating in. However, we also often scale information down, when for example looking at large-scaled models of cells or organs in educational institutions and relate information to the smaller referents. The majority of previous studies so far focused exclusively on scaling up spatial information, in which participants were presented with a small-scaled map and a larger referent space^3,5,6,8,10–12,20,21,37^.

One of the rare studies that investigated the effects of scaling directions in a sample of children found that children performed more accurately in the scaling-down condition as compared to the scaling-up condition^38^. The authors concluded that children struggled more with scaling up vs. scaling down and that different processes may be involved for these scaling directions. A more recent study from Plumert et al. (Ref.^9^, Experiment 1) supported this finding as children and adults had more difficulties with scaling up as compared to scaling down. But why could that be the case? An explanation of these findings concerns the size of the referent space in both studies. In Experiment 1 from Plumert et al.^9^, participants had to scale up information from 16 to 128 inch in the scaling-up condition. In the scaling-down condition, another sample of participants scaled spatial information down from 128 to 16 inch. Consequently, answers were given in a 128-inch large referent space in one condition, whereas participants of the other condition gave answers in a 16-inch large referent space. Similarly, Siegel et al.^38^ used a large room as referent space in the scaling-up condition and a small-scaled model as referent space in the scaling-down condition. Naturally, larger spaces give more room for errors which may explain why participants performed less accurately in the scaling-up as opposed to the scaling-down condition in the above-mentioned studies^12^. Indeed, when Plumert et al. (Ref.^9^; Experiment 3) kept size of the referent space constant across the two conditions (scaling up: 32 to 64 inches, scaling down: 128 to 64 inches), participants did no longer show larger errors for the scaling-up than the scaling-down condition. This finding is corroborated by research using a similar approach in adults^14^. Accordingly, findings of this latter study failed to show any effects of scaling direction on accuracy nor response times. In sum, results of recent studies suggest comparable effects of scaling factor on errors and response times when scaling up and scaling down. While these results seem to hold true for the visual domain, studies in the tactile domain are non-existent as of today. Building on the idea of functional equivalence of vision and touch in processing spatial information^28,30,35^, we may expect that scaling up and down in the tactile domain is performed analogously to the visual domain.

### Objectives and hypotheses

In the present study, we aimed to investigate adults’ spatial scaling strategies with a particular focus on exploring effects of perceptual modality and scaling direction, building on the theoretical models of mental imagery^19^ and the functional equivalence theory^28^. To this end, we compared adults’ spatial scaling ability in the visual and haptic domain. As we typically obtain information from several modalities simultaneously, we compared adults’ scaling performance in these single modality conditions to a combined visual and haptic perceptual condition. In this bimodal condition, participants were allowed to look at and touch the presented stimuli simultaneously. In all three perceptual conditions, we used a recently developed methodology^21^ to assess participants’ errors and response times in the scaling process. This procedure measures scaling from memory and enables to draw conclusions about the usage of spatial scaling strategies. At the same time, we filled the gap in our knowledge about the influence of scaling direction. By systematically varying the sizes of the maps that were smaller or larger than the referent space, we were able to test how adults would scale information up and down in the haptic and visual domain.

Considering that previous research suggested that participants will use mental transformation strategies for spatial scaling in the visual^8,14^ and haptic domain^20,21^, we expected systematic variations of scaling factors on participants’ errors and response times. In addition, we expected similar effects of scaling factors for each scaling direction^9,14^. Given that we used a systematic variation of scaling factor with respect to scaling up and down in a single design (i.e., 1:3, 1:2, 1:1, 2:1, 3:1), we expected the impact of scaling factor on absolute errors and response time being best described by a quadratic function for adults’ absolute errors (Hypothesis 1) and response times (Hypothesis 2). Thus, following the illustration of mental transformation strategies in Fig. 1, we expected fewer absolute errors and lower response times in the 1:1 scaling factor, in which maps and referent space had the same size. However, with increasing scaling factor in each direction, we expected higher absolute errors and response times. In line with the functional equivalence theory^28,30,35^, we did not predict differences in result patterns between the perceptual conditions.

## Methods

All procedures performed in the current study involving human participants are in accordance with the ethical standards of the institutional research committee and with the 1964 Helsinki declaration and its later amendments or comparable ethical standards (Version 2013, excluding pre-registration). The procedure received approval from the Ethical Committee of the Institute of Psychology of The John Paul II Catholic University of Lublin. All participants gave written informed consent before data collection.

### Participants

Participants were recruited among Polish students from various fields of studies via an online questionnaire promoted through social media. The study comprised a sample of 90 healthy, right-handed adults (50% female), aged between 19 and 28 years (*M* = 22.77, *SD* = 1.70) with normal or corrected-to-normal vision and without motor disabilities. One additional participant was tested but had to be excluded from the final sample because of technical problems. Among the final sample, approx. 19% of the participants (*n* = 17) were psychology students. All participants were tested in the haptic, visual, and visuo-haptic condition, with the order of the perceptual conditions being counterbalanced across participants (and across gender). Thus, participants were randomly assigned to six groups with different orders (*n* = 15 per group). A priori power analyses (using G-Power 3.1^39^) based on a moderate effect size of *f* = 0.25^10^, using significance levels of *p* < 0.05, and a power of 0.80 showed that a minimum sample size of 45 participants is required to detect a within-participant effect in a repeated measures analysis of variance (ANOVA). Due to a potential influence of the between-participants variable of order as observed in previous research^21^, we opted to test more participants, and doubled the sample size. Participants were compensated with 50 PLN (approx. 11 Euro) for their participation.

### Materials

Participants were presented with a flat wooden-like board which had the same size for all trials. This wooden-like board contained a black rectangular map made of felt, including a round colored target made of a linoleum-like material and thus, a contrasting material. Therefore, embossed parts of the map (e.g., the map surface and target) were made of different textures. Recent studies investigating the perception of tactile graphics^40–42^ revealed that graphics produced in such a “collage” technique/textured pictures may be easier to interpret for blindfolded participants as opposed to a raised-line technique. Therefore, we opted to use tactile graphics produced in this “collage” technique as combining sensations from different materials with convex elements seems to improve participants’ encoding of embossed graphics^42^.

Participants were asked to learn the location of the pink target and reproduce it in an empty referent space by locating a round, convex response object of the same pink color (15 mm in diameter). The referent space had a constant size (30 mm × 90 mm) throughout the trials. In order to manipulate the scaling factors (SFs), maps had five different sizes: 10 mm high × 30 mm wide (SF = 1:3), 15 mm high × 45 mm wide (SF = 1:2), 30 mm high × 90 mm wide (SF = 1:1), 60 mm high × 180 mm wide (SF = 2:1), and 90 mm high × 270 mm wide (SF = 3:1). The target was placed in five equidistant locations on the map and was varied on the horizontal dimension of the map while the position on the vertical location was held constant across the trials (i.e., the target was always centered on this vertical dimension). The target diameter was scaled in the same way as the rest of the spatial layout. Like the target, maps and referent spaces were convex as well and overall size could be easily explored by touch. Scaling factors and target locations were combined using a full-factorial design, amounting to a total of 25 rectangular maps with each one presenting a single target location^6,8,11,12^ (see Supplementary Methods 1 for detailed information on the stimuli’s sizes and coordinates and Supplementary Methods 2 for photographs of the stimuli).

### Procedure

Participants were tested individually in a single test session, including the spatial scaling task. Then, the participants were asked about demographic details (gender, age, field of study), experience with using maps, and interest in spatial tasks.

In the spatial scaling task, each participant learned the position of the target (a) visually (V), (b) haptically (H) and (c) visuo-haptically (VH). Order of these perceptual learning conditions were pseudo-randomized (using six different orders) across participants. Each perceptual learning condition was preceded by a corresponding short training to familiarize participants with the visual, haptic or visuo-haptic encoding of the targets. Sizes of the training and response object were kept constant, but coordinates did not correspond to the ones presented in the test phase. The main task comprised 25 test trials per each perceptual condition presented in random order. Thus, each participant solved a total of 75 test trials. Two experimenters worked together in order to carry out the spatial scaling task. In the visual and visuo-haptic conditions, participants were asked to close their eyes and open them only when the experimenter said “now”. In the haptic condition, participants were blindfolded prior to this condition. Each trial accorded to the following procedure: one experimenter placed the wooden-like board with the map on a table in front of the participant and said “now” so that participants could open their eyes and/or touch the board to encode the target’s location on the map. Once participants signaled that they had learned the location and closed their eyes, the experimenter removed the map. Then, the other experimenter placed the referent space together with the response object in front of the participant (see Supplementary Methods S2). The response object was always placed to the right of the referent space on the board. At the same time, the experimenter said “now” to indicate that participants may open their eyes (in the visual and visuo-haptic condition) or touch the space (in the haptic condition). Participants reproduced the target’s location from the previous map on the referent space and said “now” to indicate their answer. The experimenter then took a picture of the board (at a fixed resolution). Immediately afterwards, the next trial began by asking participants to close their eyes in both visual conditions and wait for the next map, while blindfolded participants in the haptic condition were informed verbally. The time for learning the map and giving the answer in the referent space was measured by the experimenters using stop timers (each of the experimenters measured one type of time, either learning or response times). After each trial, the experimenter took a photo of the referent space including the participant’s response. A script written in Python programming language analyzed each photograph by finding the center of the response object and the coordinates of the reference space and assessed the x- and y-coordinates of the participant’s response.

### Data preparation

#### Reversal errors

Previous research has shown that participants occasionally produce reversal errors in which they locate responses on the wrong side of the referent space^5,8–11^. In the current study, we checked for these reversal errors. In line with previous studies^5,8–11,21^, we coded responses given on the right side of the board (i.e., x-coordinate of the response > 45 mm) as reversal errors when targets were originally presented of the left side of the map (as seen from the midpoint). Accordingly, responses given on the left side of the board (i.e., x-coordinate of the response < 45 mm) were coded as reversal errors when targets were originally presented on the right side of the map. To investigate whether these reversal errors occurred systematically, we computed an ANOVA with the within-participant variables scaling factor and perceptual condition. Based on previous studies^8,11^, we did not expect any effects of scaling factor on participants’ propensity to commit such reversal errors, but explored effects of perceptual condition.

#### Absolute errors

As a general index for adults’ accuracy in locating the targets, we used the absolute errors as reflected by the Euclidean distance between a participant’s response and the correct target location. This distance was calculated automatically based on the x- and y-coordinates by a program written in Python programming language for this current research study. Given that the reversal errors produced large variability in the data and did not covary with any variable of interest in participants, we used a corrected version of these absolute errors similar to previous research (e.g., Ref.^9^). That is, in case of reversal errors, participants’ responses are coded as if the target was located on the correct side on the horizontal dimension (i.e., the x-coordinate of the target on a respective target location from the participant’s response). Importantly, analyses with uncorrected data revealed similar effects with respect to the variables of interest on participants’ absolute errors (i.e., scaling factor, perceptual condition).

#### Learning times

Learning times reflect the phase 1 of the scaling task. They were measured from the moment participants started exploring the map either haptically, visually, or visuo-haptically, to the moment they signaled being ready to proceed and locate the target on a map (phase 2 of the scaling task).

#### Response times

Response times were measured from the moment the experimenter placed the referent space in front of the participants (indicated to the participant by saying “ready”) to the moment the participants said “ready” as a signal that they had placed the response disc on the map. As participants got to know the referent space in this phase and scaled spatial information accordingly, these response times are assumed to reflect the spatial scaling process. Even though these response times also include exploring the referent space and locating the target, variations most likely stem from the scaling process given that the referent space is constant-sized across trials and the location procedure should be comparable across trials.

#### Outliers

We identified and excluded outliers (*M* ± 3 *SD*s) in participants’ absolute errors, signed errors, learning times and response times. The outliers comprised 1.72% of all cases (from a total of 6750 responses) with respect to absolute errors, 2.30% cases of the learning times and in 2.16% cases of the response times. Excluding outliers caused two participants with extremely high learning times to be excluded (hence, *n* = 88 when analysing learning times), and one participant with extremely high response times to be excluded (hence, *n* = 89 when analysing response times). Data were collapsed across all trials of each participant, but separately for each perceptual condition (haptic, visual and visuo-haptic) and each scaling factor (1:3, 1:2, 1:1, 2:1, 3:1).

### Statistical approach

We conducted a 3 × 5 × 6 repeated measures ANOVA with perceptual condition (haptic vs. visual vs. visuo-haptic) and scaling factor (1:3 vs. 1:2 vs. 1:1 vs. 2:1 vs. 3:1) as within-subject factors and perceptual order (H-V-VH vs. H-VH-V vs. V-H-VH vs. V-VH-H vs. VH-V-H vs. VH-H-V) as a between-subject factor for the following dependent variables: reversal errors, absolute errors, signed errors, learning times, and response times. We were specifically interested in seeing differences between perceptual conditions. Furthermore, we examined whether dependent variables differed as a function of scaling factor and checked which contrast in the ANOVA would explain data patterns best. Furthermore, we explored the symmetry of these data patterns by comparing performance for congruent scaling factors (1:3 vs. 3:1; 1:2 vs. 2:1) in order to see whether scaling directions (up vs. down) would influence participants’ scaling performance.

## Results

### Reversal errors

Participants sometimes produced reversal errors, in which they located the target on the other half of the referent space (12.18% of all cases). At the same time, there were no significant main or interaction effects (see Table 1), showing that the propensity of committing reversal errors was not related to scaling factor, perceptual condition, nor order of perceptual condition.

**Table 1:** Results of the ANOVA conducted for the reversal errors and absolute errors.

	Reversal errors				Absolute errors			
	*dfs*	*F*	*p*	η_p_^2^	*dfs*	*F*	*p*	η_p_^2^
Perceptual condition	1.81, 151.77	0.64	0.513	0.01	**1.75, 147.27**	**75.91**	** < 0.001**	**0.48**
Scaling factor	3.29, 276.50	0.60	0.628	0.01	**3.43, 288.01**	**3.87**	**0.007**	**0.04**
Order of perceptual presentation	5, 84	0.38	0.865	0.02	**5, 84**	**12.11**	** < 0.001**	**0.42**
Perceptual condition × order of perceptual condition	9.03, 151.77	1.41	0.189	0.08	8.77, 147.27	1.55	0.137	0.09
Scaling factor × order of perceptual condition	16.46, 276.50	1.22	0.247	0.07	17.14, 288.00	0.53	0.94	0.03
Scaling factor × perceptual condition	6.39, 536.35	1.63	0.131	0.02	6.78, 569.35	0.64	0.714	0.01
Scaling factor × perceptual condition × order of perceptual presentation	31.93, 536.35	0.94	0.568	0.05	33.89, 569.35	1.28	0.137	0.07

Significant effects are marked in bold.

### Absolute errors

**Figure 2:**
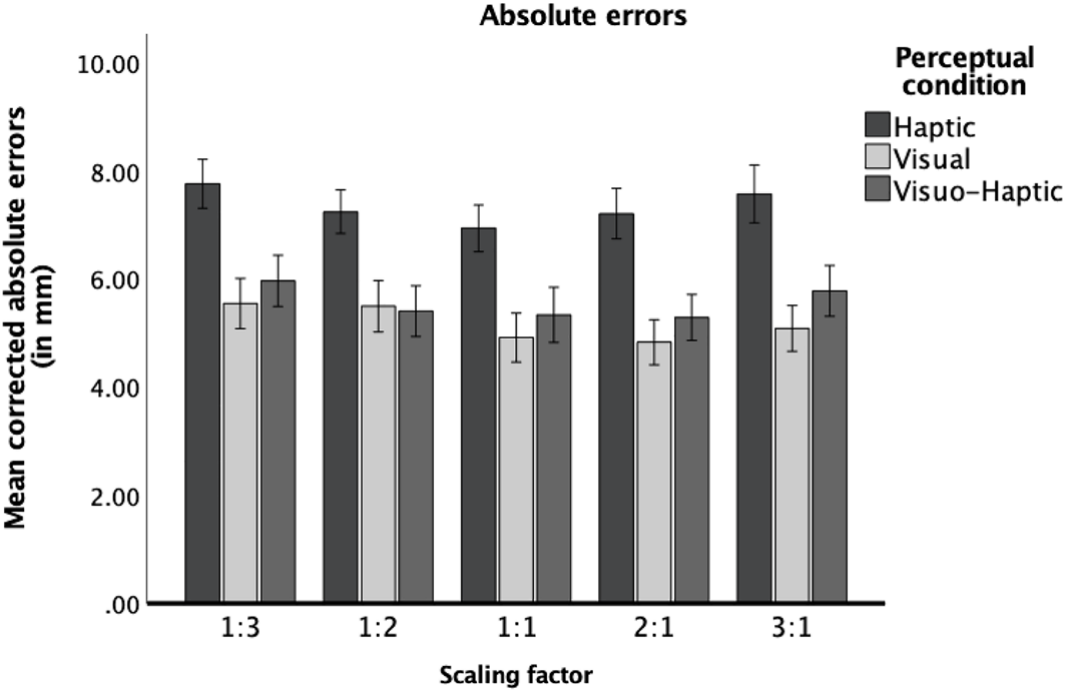
Mean absolute errors presented for each scaling factor in each perceptual condition. Error bars stand for ± 1 SE.

The ANOVA revealed a significant main effect of perceptual condition (for inferential statistics, see Table 1). Post-hoc pairwise comparisons (with Bonferroni correction here and throughout) showed that participants produced larger errors in the haptic than in the visual and visuo-haptic conditions (*p* < 0.001 for both comparisons, for descriptive statistics, see Supplementary Table S1). The difference between visual and visuo-haptic conditions was nonsignificant (*p* = 0.058). There was also a significant main effect of scaling factor, which was best explained by a quadratic function, *F*(1,84) = 10.76, *p* = 0.002, η_p_^2^ = 0.11. As indicated by the nonsignificant interaction between scaling factor and perceptual condition, participants produced larger errors for higher scaling factors (1:3 and 3:1) as opposed to smaller scaling factors (1:2 and 2:1), regardless of the perceptual condition (see Fig. 2). Post-hoc pairwise comparisons did not yield significant differences in accuracy between conditions of 3:1 and 1:3 (*p* = 1.00) nor 2:1 and 1:2 (*p* = 1.00). This suggests that scaling direction does not seem to influence spatial scaling accuracy. The main effect of perceptual order was also significant (for descriptive statistics, see Supplementary Table S2; significant differences are further presented in Supplementary Fig. S1). It seems that particularly those participants who experienced the visual conditions after the haptic experience produced fewer absolute errors.

### Learning times

The analysis yielded a significant main effect of perceptual condition (for inferential statistics, see Table 2; for descriptive statistics, see Supplementary Table S3). Follow-up comparisons revealed that learning took the longest time in the haptic condition (*p* < 0.001 for both comparisons with the visual and visuo-haptic condition) and was the fastest in the visual condition (*p* < 0.001, for the comparison with the visuo-haptic condition).

**Table 2:** Results of the ANOVA conducted with the learning times and response times.

	Learning times				Response times			
	*dfs*	*F*	*p*	η_p_^2^	*dfs*	*F*	*p*	η_p_^2^
Perceptual condition	**1.42, 116.30**	**173.92**	** < 0.001**	**0.68**	**1.34, 110.95**	**133.35**	** < 0.001**	**0.62**
Scaling factor	**1.98, 161.94**	**46.73**	** < 0.001**	**0.36**	**3.06, 254.20**	**12.77**	** < 0.001**	**0.13**
Order of perceptual presentation	5, 82	1.61	0.166	0.09	5, 83	1.64	0.159	0.09
Perceptual condition × order of perceptual condition	**7.09, 116.30**	**2.86**	**0.008**	**0.15**	6.68, 110.95	1.44	0.198	0.08
Scaling factor × order of perceptual condition	9.87, 161.94	1.62	0.106	0.09	15.31, 254.20	1.37	0.161	0.08
Scaling factor × perceptual condition	**4.44, 363.78**	**14.29**	** < 0.001**	**0.15**	5.54, 460.27	0.98	0.433	0.01
Scaling factor × perceptual condition × order of perceptual presentation	22.18, 363.78	0.78	0.756	0.05	27.73, 460.27	0.99	0.477	0.06

Significant effects are marked in bold.

**Figure 3:**
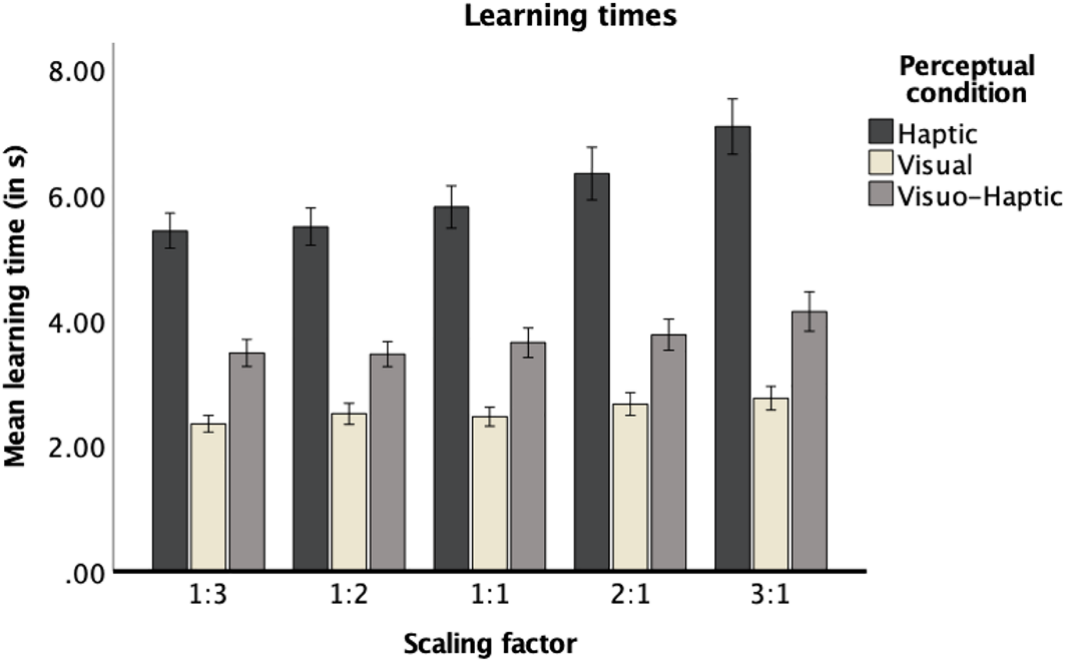
Learning times presented for each scaling factor in each perceptual condition. Error bars stand for ± 1 SE.

The main effect of scaling factor was significant, but this effect was qualified by a significant interaction between scaling factor and perceptual condition. Separate ANOVAs conducted for each perceptual condition showed a significant main effect of scaling factor, *F*(2.39, 195.91) = 36.98, *p* < 0.001, η_p_^2^ = 0.31, for the haptic condition, *F*(2.80, 228.69) = 12.27, *p* < 0.001, η_p_^2^ = 0.13, for the visual condition, and *F*(2.28, 187.23) = 14.51, *p* < 0.001, η_p_^2^ = 0.15, for the visuo-haptic condition. The pattern of results was best explained by a linear function in each perceptual condition, *F*(1, 82) = 60.14, *p* < 0.001, η_p_^2^ = 0.42, for the haptic condition, *F*(1, 82) = 28.67, *p* < 0.001, η_p_^2^ = 0.26, for the visual condition, and *F*(1, 82) = 27.31, *p* < 0.001, η_p_^2^ = 0.25, for the visuo-haptic condition. As can be seen in Fig. 3, the interaction can be explained by the different slopes across the perceptual conditions: participants produced higher learning times with increasing size of the maps, which seems especially prominent in the haptic condition.

The same ANOVA also revealed a significant interaction between perceptual condition and perceptual order (see Supplementary Table S4, for detailed descriptive statistics). This interaction may stem from participants’ lower learning times in the haptic condition when being presented with the visual conditions prior to the haptic condition.

Similarly, the interaction between scaling factor and perceptual order was significant. Separate ANOVAs conducted for each of the six “perceptual order” groups yielded a significant main effect of scaling factor for each perceptual order. However, in the H-VH-V group, the pattern of results was best explained by the quadratic function whereas in the other groups the linear function was the best fit (see Supplementary Table S5, for the detailed results of the separate ANOVAs and contrast effects; see Supplementary Table S6, for the descriptive statistics).

### Response times

**Figure 4:**
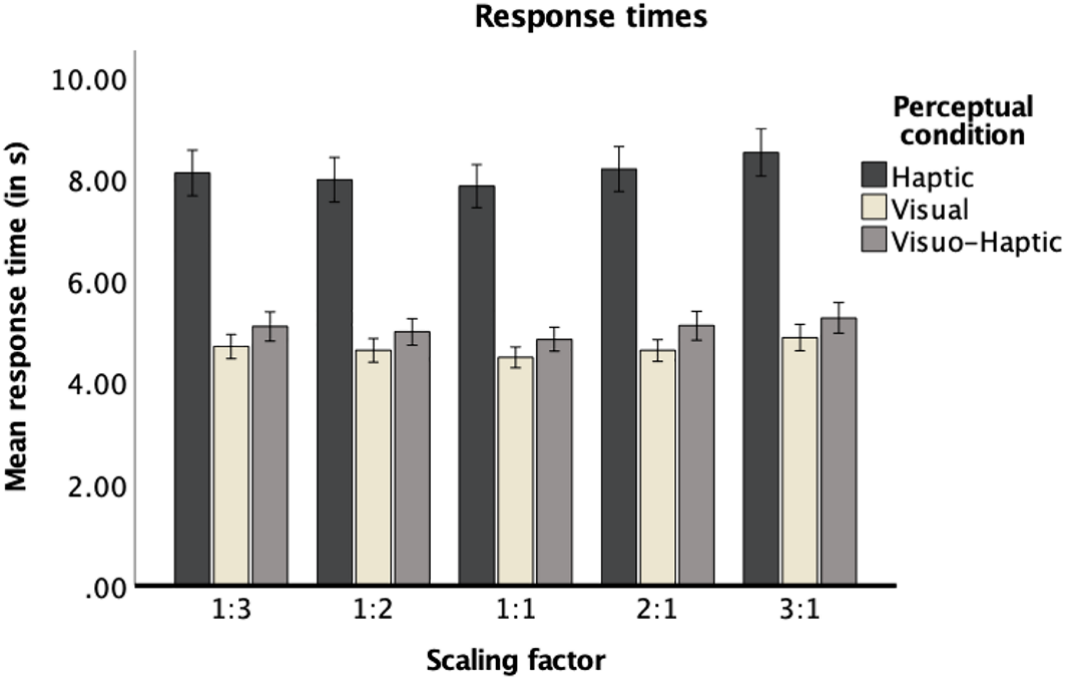
Response times for each scaling factor in each perceptual condition. Error bars stand for ± 1 SE.

The ANOVA yielded a significant main effect of perceptual condition (for inferential statistics, see Table 2). Pairwise comparisons revealed that responses times were the highest in the haptic condition (*p* < 0.001, for comparisons with the visual and visuo-haptic condition) and were the shortest in the visual condition (*p* < 0.020, for the comparison with the visuo-haptic condition). The main effect of scaling factor was also significant and was best explained by a quadratic function, *F*(1, 83) = 19.44, *p* < 0.001, η_p_^2^ = 0.19 (for the descriptive statistics, see Supplementary Table S7). As indicated by the nonsignificant interaction between scaling factor and perceptual condition, participants showed longer response times for higher scaling factors (1:3 and 3:1) as opposed to smaller scaling factors (1:2 and 2:1), regardless of perceptual condition (see Fig. 4). Post-hoc pairwise comparisons did not yield significant differences in the response times between scaling factors conditions of 2:1 and 1:2 (*p* = 1.00), but response times were larger for 3:1 than 1:3 (*p* = 0.001; see Supplementary Table S7). This suggests that scaling down might be more time-consuming than scaling up.

## Discussion

In the current study, we aimed to test adults’ strategies in spatial scaling from memory in different perceptual domains, using a recently developed methodology^21^. To this end, we compared response times and absolute errors in a spatial scaling task performed in the visual and haptic domain and a bimodal condition. This procedure allows assessing the spatial scaling strategies adopted by participants (absolute, relative distances or mental transformation)^13,14^. In addition, we assessed whether scaling direction would influence spatial scaling ability, and included scaling factors that required both scaling up (1:3, 1:2) and down (2:1, 3:1), compared to the control condition (1:1). Considering that previous studies focused mainly on scaling up, i.e., comparing smaller maps with larger referent spaces^3,5,6,8,10–12,20,21,37^), this design helped increasing our knowledge about the flexibility of spatial scaling in these two directions.

In general, our hypotheses regarding the scaling strategy adopted by the participants were supported. We found that the scaling factor influenced both absolute errors and response times when performing the localization task. Importantly, both effects were best described by a quadratic function. Since we used a systematic variation of scaling factor with respect to scaling up and down within a single design, these findings suggest that participants used mental transformation strategies when scaling spatial layouts in both directions. Thus, our results echo findings of a previous adult study that used a discrimination task and showed the usage of mental transformation strategies irrespective of scaling direction (up versus down)^14^. Besides, our findings suggested that mental transformation strategies seemed to be adopted regardless of the visual or haptic domain. In other words, updating spatial layouts in terms of size was similarly processed in the visual and haptic domains, supporting the idea of functional equivalence of spatial images from touch and vision^28,30,35^. Therefore, our findings are in line with previous research showing the adoption of mental transformation strategies in the visual or haptic domain^8,14,20,21^.

Although participants were similarly prone to produce higher absolute errors with increasing scaling factors in each perceptual condition, spatial scaling in perceptual domains differed significantly. Spatial scaling accuracy was higher in both visual conditions (i.e., visual and bimodal) than in the haptic condition. At the same time, learning times and response times were higher in the haptic condition than compared to both visual conditions. Moreover, as the difference between visual and bimodal conditions was nonsignificant, the possibility of an additional haptic perception of maps did not contribute to a more accurate performance in localizing the disc. However, it took participants longer to learn the map in the visuo-haptic condition than in the visual one and likewise, response times were longer in the bimodal condition than in the visual condition. These patterns of results may indicate a lower effectiveness of haptic than visual perception of spatial stimuli^22–25^. Previous studies using tasks that required a mental transformation of size have also shown that spatial cognition is more accurate in the visual domain than the haptic domain^21,36,37^. A similar effect was found in studies on a canonical size phenomenon tested through a drawing task. Although the canonical size effect was found for both visual and tactile modalities, the quality of drawings made under visual control was higher than those produced while being blindfolded^34^.

Our research contributes to a better understanding of the effects of scaling direction on spatial scaling accuracy, filling a knowledge gap on this topic in the haptic domain. Results revealed that regardless of modality, scaling up is not more difficult than scaling down as was suggested by Siegel et al.^38^. More specifically, participants did not produce larger errors for scaling factors 1:3 than 3:1 nor 1:2 than 2:1. These findings align with findings yielded for spatial scaling in the visual domain^9,14^). At the same time, the pattern of results was slightly different concerning response times. For the largest scaling factors (3:1 and 1:3), it took participants longer to give the response when scaling down whereas this held not true for smaller scaling factors such as 2:1 and 1:2. A previous study that considered the direction of change in object size in an imagery task and used the same scaling factors as in the current study has not shown a similar effect on response times^36^. Hence, this finding needs further exploration and replication.

Our analyses also yielded several effects in addition to our hypotheses. First, participants produced reversal errors on several occasions. Such errors were also found in previous studies investigating adults’ spatial scaling, especially in the haptic domain (e.g.^21^). These reversal errors indicate that participants confused the right and left side of the board and thus, suggest that even adults were challenged from time to time in our task. Second, the between-subjects factor of perceptual order has influenced participants’ absolute errors, with those participants who experienced the visual conditions after the haptic experience producing fewer absolute errors. However, this main effect seems rather arbitrary and is hardly interpretable. Our findings also suggested an interaction between perceptual order and perceptual condition on participants’ learning times. This interaction may stem from participants’ quicker learning times in the haptic condition when being presented with the visual conditions prior to the haptic condition. This could be interpreted such that participants who saw the boards with maps at earlier stages of the experimental session may have had an easier time visualizing them while performing the task under the haptic condition, which resulted in accelerated learning.

### Strengths, limitations and suggestions for future research

In our opinion, the strengths of our study refer to using a recently developed methodology that allows assessing specific spatial scaling strategies in various perceptual modalities^21^. Additionally, our experimental design allowed measuring effects of different scaling directions. Up to date, studies considering both scaling directions have been rare^9,14,38^ and only tested spatial scaling in the visual domain^3,5,6,8,10–12,20,21,37^.

In addition to these strengths, several limitations warrant mention. We consider it a limitation that target distribution on the map varied only on the horizontal dimension. Thus, our findings could be interpreted as showing that mental transformation strategies were used in relatively low demanding conditions. Future studies should test whether mental transformation strategies are also used when scaling maps in more demanding two-dimensional conditions (i.e., when targets on the maps varied on horizontal and vertical dimensions, see, e.g.^1,11^, for a similar design). Combining dimensionality and perceptual domains would allow to investigate whether spatial scaling is less accurate in the haptic than the visual domain when using more complex maps (see^30^).

Furthermore, our results were only based on a relatively homogenous sample of young adults. Future studies might be conducted with other groups of participants, for example, children and individuals with blindness. We know little about the influence of scaling direction on spatial scaling among children^9,38^, and the developmental progression in this ability has not been investigated in the haptic domain. Investigating such a developmental progression seems especially interesting given that the functional equivalence of vision and haptics might be age-dependent as found in a previous study on shape recognition^43^. Therefore, future research may explore spatial scaling in different perceptual domains and in various age groups (children, adolescents, younger adults, and older adults) and systematically compare functional equivalence among these groups. Moreover, to our knowledge, participants who are blind have only been tested in the scaling up condition^11^. It seems crucial to examine blind participants, as spatial scaling ability seems necessary for their daily functioning, when for example blind adults navigate independently through a city using a map.

Another factor that might be investigated in future research refers to systematically varying delay intervals between memorization (learning a map) and test phases (responding in the referent space)^43^. Finally, it might be important to assess the concrete exploration strategies when learning about the map. Future studies may investigate and classify such strategies in order to assess their differential effects on participants’ scaling performance. By doing so, it might be interesting to code in detail whether participants focused on specific components of the map (i.e., the target, borders) to scale spatial information accordingly.

## Conclusions

Overall, the current study on spatial scaling from memory supports the notion that, like in other imagery tasks^16–19^, mental imagery strategies are adopted in spatial scaling. Notably, adults seemed to use mental transformation strategies, irrespective of the perceptual domain (i.e., haptic vs. visual) and scaling direction (up vs. down).

### Supplementary Information

Supplementary Information.

## Data Availability

The datasets generated and analyzed during the current study are available in the figshare repository (10.6084/m9.figshare.22303162).
