# Supplementary material for: Effects of scaling direction on adults’ spatial scaling in different perceptual domains
Source: Sci Rep. 2023 Sep 6;13:14690. doi: 10.1038/s41598-023-41533-3 (PMC10482972; doi:10.1038/s41598-023-41533-3)
Supplement: Supplementary file 1 — Supplementary Information. [file 41598_2023_41533_MOESM1_ESM.pdf]

Supplementary Methods 1. Characteristics of the materials used in the study: Stimuli sizes and coordinates of the targets.

**Scaling factor**	**Target diameter [mm]**	**Map sizes (dimensions of the black rectangle) [mm]**		**Coordinates of the target position [mm]**		**Map number**
		**OX**	**OY**	**OX**	**OY**	
**1:3**	5	30	10	5	5	1
				10	5	2
				15	5	3
				20	5	4
				25	5	5
**1:2**	7.5	45	15	7.5	7.5	6
				15	7.5	7
				22.5	7.5	8
				30	7.5	9
				37.5	7.5	10
**1:1**	15	90	30	15	15	11
				30	15	12
				45	15	13
				60	15	14
				75	15	15
**2:1**	30	180	60	30	30	16
				60	30	17
				90	30	18
				120	30	19
				150	30	20
**3:1**	45	270	90	45	45	21
				90	45	22
				135	45	23
				180	45	24
				225	45	25

Supplementary Methods 2: Photographs of the exemplar materials used in the study.

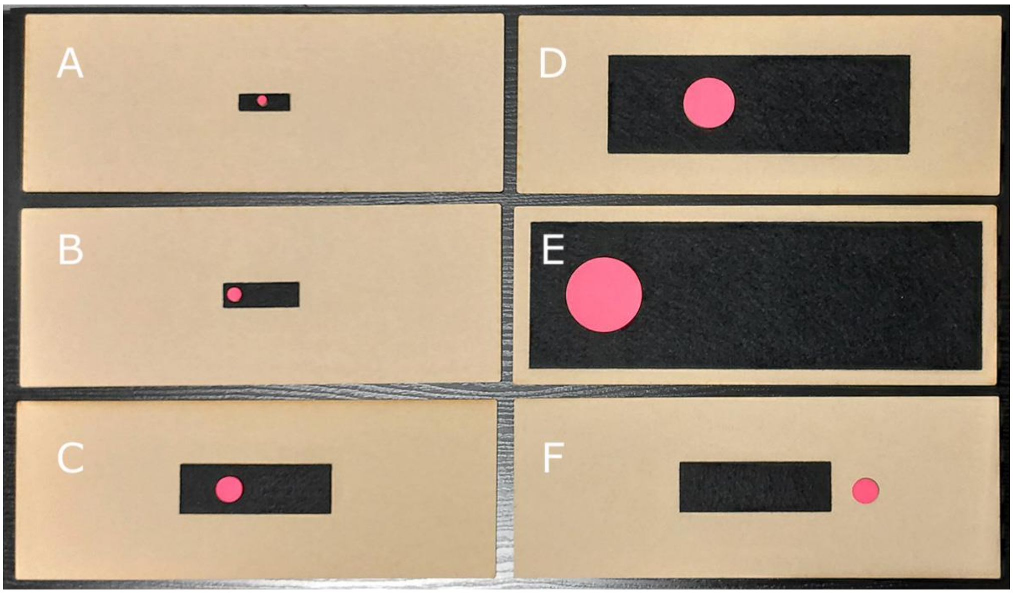

Supplementary Figure S1. Mean absolute errors (in mm) presented separately for each group of the perceptual presentation. Error bars stand for +/- 1 SD.

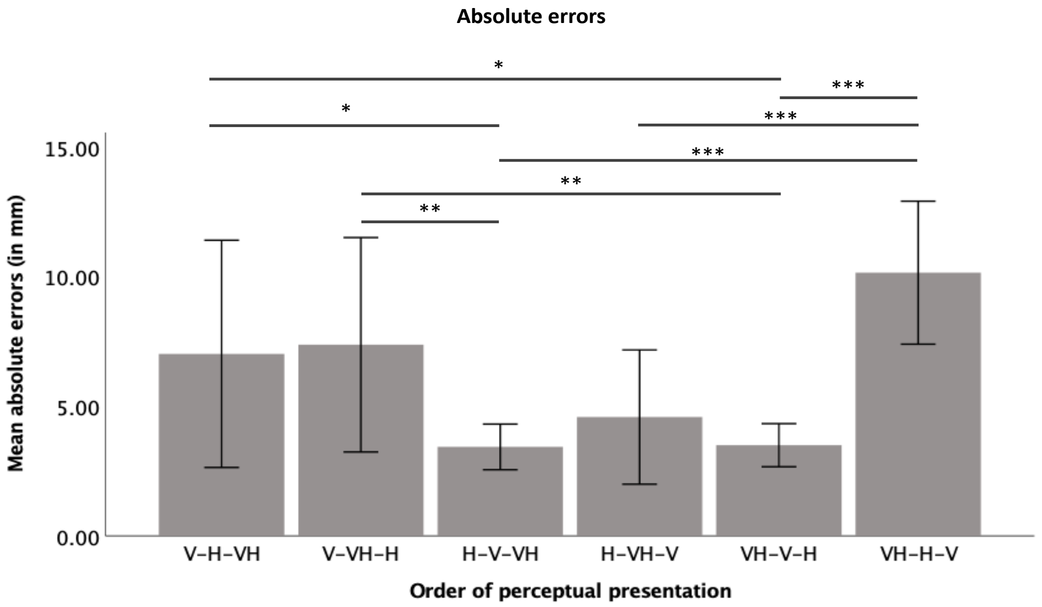

Note. * p < .05, ** p < .01, *** p < .001. V = Visual, H = Haptic, VH = Visuo-Haptic.

Supplementary Table S1. Descriptive statistics (means with standard deviations in parentheses) of the absolute errors (in mm) presented for each level of the scaling factor and separately for each perceptual condition.

**Perceptual condition**	**Scaling Factor**					
	**1:3**	**1:2**	**1:1**	**2:1**	**3:1**	**TOTAL**
Haptic	7.75	7.23	6.92	7.20	7.56	**7.33**
	(4.27)	(3.88)	(4.16)	(4.47)	(5.08)	**(4.37)**
Visual	5.53	5.48	4.90	4.82	5.07	**5.16**
	(4.40)	(4.51)	(4.25)	(3.92)	(4.00)	**(4.22)**
Visuo-haptic	5.94	5.39	5.32	5.27	5.76	**5.54**
	(4.50)	(4.45)	(4.82)	(4.03)	(4.45)	**(4.45)**
**Total**	**6.41**	**6.03**	**5.71**	**5.76**	**6.13**	**6.01**
	**(4.39)**	**(4.28)**	**(4.41)**	**(4.14)**	**(4.51)**	**(4.35)**

Supplementary Table S2. Descriptive statistics (means and standard deviations) of the absolute errors (in mm) presented separately for each group with different order of perceptual presentation.

**Order of perceptual presentation**	**Absolute errors**	
	M	***SD***
H-V-VH	3.43	0.88
H-VH-V	4.58	2.59
V-H-VH	7.02	4.38
V-VH-H	7.37	4.13
VH-V-H	3.50	0.83
VH-H-V	10.15	2.76

Note. V = Visual, H = Haptic, VH = Visuo-Haptic.

Supplementary Table S3. Descriptive statistics (means with standard deviations in parentheses) of the learning times (in s) presented for each level of the scaling factor and for each perceptual condition.

**Perceptual condition**	**Scaling factor**					
	**1:3**	**1:2**	**1:1**	**2:1**	**3:1**	**TOTAL**
Haptic	5.43	5.49	5.81	6.34	7.09	**6.03**
	(2.69)	(2.84)	(3.17)	(3.92)	(4.12)	**(3.35)**
Visual	2.35	2.51	2.46	2.66	2.75	**2.55**
	(1.24)	(1.58)	(1.42)	(1.71)	(1.78)	**(1.54)**
Visuo-haptic	3.47	3.46	3.64	3.76	4.14	**3.69**
	(2.02)	(1.88)	(2.19)	(2.32)	(2.94)	**(2.27)**
**Total**	**3.75**	**3.82**	**3.98**	**4.27**	**4.66**	**4.09**
	**(1.98)**	**(2.10)**	**(2.26)**	**(2.65)**	**(2.95)**	**(2.39)**

Supplementary Table S4. Descriptive statistics (means and standard deviations in parentheses) of the learning times (in s) presented for each group of the perceptual order factor, separately for each perceptual condition.

**Perceptual**	**Order of perceptual presentation**					
**condition**	V-H-VH	V-VH-H	H-V-VH	H-VH-V	VH-V-H	VH-H-V
Haptic	4.17	4.95	7.27	6.74	6.37	6.76
	(1.22)	(2.99)	(4.09)	(4.05)	(2.40)	(3.23)
Visual	1.90	2.21	2.89	2.95	2.78	2.58
	(0.73)	(1.27)	(2.25)	(1.96)	(0.85)	(1.32)
Visuo-	2.72	3.43	3.61	3.79	4.85	3.83
haptic	(0.96)	(2.51)	(2.58)	(2.58)	(1.91)	(2.13)

Note. V = Visual, H = Haptic, VH = Visuo-Haptic.

Supplementary Table S5. Results of the analyses of variance conducted separately for each perceptual order group.

Order of perceptual presentation	Main effect of scaling factor				Best explaining function				
	dfs	***F***	***p***	η_p^2	**type**	***dfs***	***F***	***p***	η_p^2
V-H-VH	4, 56	8.02	<.001	.36	linear	1, 14	14.74	.002	.51
V-VH-H	1.97, 27.59	5.19	.013	.27	linear	1, 14	8.69	.010	.38
H-V-VH	1.45, 20.30	8.76	.004	.39	linear	1, 14	12.26	.004	.47
H-VH-V	1.39, 18.09	8.13	.006	.39	quadratic	1, 13	12.06	.004	.48
VH-V-H	1.83, 23.84	11.43	<.001	.47	linear	1, 13	18.84	<.001	.60
VH-H-V	1.76, 24.67	8.94	.002	.39	linear	1, 14	13.45	.003	.49

Note. V = Visual, H = Haptic, VH = Visuo-Haptic.

Supplementary Table S6. Descriptive statistics (means and standard deviations in parentheses) of the learning times (in s) presented for each group of the perceptual order factor, separately for each scaling factor.

**Order of perceptual presentation**	**Scaling factor**				
	**1:3**	**1:2**	**1:1**	**2:1**	**3:1**
V-H-VH	2.79	2.83	2.86	2.92	3.25
	(0.70)	(0.85)	(0.90)	(0.93)	(1.03)
V-VH-H	3.33	3.40	3.43	3.67	3.82
	(1.82)	(2.16)	(2.22)	(2.52)	(2.39)
H-V-VH	4.18	4.29	4.47	4.87	5.14
	(2.44)	(2.67)	(2.70)	(3.44)	(3.33)
H-VH-V	4.05	4.08	4.32	4.60	5.43
	(2.01)	(2.21)	(2.53)	(2.51)	(3.57)
VH-V-H	4.15	4.30	4.67	5.00	5.43
	(1.44)	(1.34)	(1.48)	(1.87)	(2.22)
VH-H-V	4.04	4.06	4.33	4.55	5.00
	(1.86)	(1.81)	(1.97)	(2.43)	(2.54)

Note. V = Visual, H = Haptic, VH = Visuo-Haptic.

Supplementary Table S7. Descriptive statistics (means with standard deviations in parentheses) of the response times (in s) presented for each level of the scaling factor, separately for each perceptual condition.

**Perceptual condition**	**Scaling Factor**					
	**1:3**	**1:2**	**1:1**	**2:1**	**3:1**	**TOTAL**
Haptic	8.12	7.98	7.86	8.20	8.52	**8.14**
	(4.23)	(4.14)	(3.99)	(4.16)	(4.36)	**(4.18)**
Visual	4.69	4.62	4.48	4.62	4.87	**4.66**
	(2.23)	(2.19)	(1.91)	(2.03)	(2.44)	**(2.16)**
Visuo-haptic	5.09	4.98	4.84	5.10	5.26	**5.05**
	(2.71)	(2.45)	(2.22)	(2.67)	(2.82)	**(2.57)**
**Total**	**5.97**	**5.86**	**5.73**	**5.97**	**6.22**	5.95
	**(3.06)**	**(2.92)**	**(2.71)**	**(2.95)**	**(3.21)**	(2.97)
